# Layer-Specific fMRI Reflects Different Neuronal Computations at Different Depths in Human V1

**DOI:** 10.1371/journal.pone.0032536

**Published:** 2012-03-20

**Authors:** Cheryl A. Olman, Noam Harel, David A. Feinberg, Sheng He, Peng Zhang, Kamil Ugurbil, Essa Yacoub

**Affiliations:** 1 Center for Magnetic Resonance Research, Department of Radiology, University of Minnesota Medical School, Minneapolis, Minnesota, United States of America; 2 Department of Psychology, University of Minnesota, Minneapolis, Minnesota, United States of America; 3 Department of Neurosurgery, University of Minnesota, Minneapolis, Minnesota, United States of America; 4 Advanced MRI Technologies, Sebastopol, California, United States of America; Cuban Neuroscience Center, Cuba

## Abstract

Recent work has established that cerebral blood flow is regulated at a spatial scale that can be resolved by high field fMRI to show cortical columns in humans. While cortical columns represent a cluster of neurons with similar response properties (spanning from the pial surface to the white matter), important information regarding neuronal interactions and computational processes is also contained within a single column, distributed across the six cortical lamina. A basic understanding of underlying neuronal circuitry or computations may be revealed through investigations of the distribution of neural responses at different cortical depths. In this study, we used T_2_-weighted imaging with 0.7 mm (isotropic) resolution to measure fMRI responses at different depths in the gray matter while human subjects observed images with either recognizable or scrambled (physically impossible) objects. Intact and scrambled images were partially occluded, resulting in clusters of activity distributed across primary visual cortex. A subset of the identified clusters of voxels showed a preference for scrambled objects over intact; in these clusters, the fMRI response in middle layers was stronger during the presentation of scrambled objects than during the presentation of intact objects. A second experiment, using stimuli targeted at either the magnocellular or the parvocellular visual pathway, shows that laminar profiles in response to parvocellular-targeted stimuli peak in more superficial layers. These findings provide new evidence for the differential sensitivity of high-field fMRI to modulations of the neural responses at different cortical depths.

## Introduction

There is recent evidence that neuronal computations related to object perception involve primary visual cortex (V1) [1], [2], [3], even though the neurons at this first stage of cortical visual processing respond only to small regions of the visual field. The mechanisms by which V1 is involved in higher order visual processing are unclear, especially given that perception of image structure can result in either increases [4] or decreases [5] of the V1 fMRI response. Because feed-forward, feed-back and local connections in V1 are segregated according to cortical depth [6], measurements of layer-specific fMRI responses should help constrain current hypotheses about the sources of these neuromodulatory effects in V1.

When functional MRI is performed at high magnetic fields, the feasibility of layer-specific investigations in human subjects improves due to the increases in both spatial resolution and signal specificity [7], [8], [9], [10]. To date, although layer-dependent activation profiles have been shown in animals and humans using fMRI [11], [12], [13], [14], [15], [16], these studies report a laminar profile measured in response to a single stimulus versus no stimulus. Further, these previous studies [13], [14], [17] acknowledge that these changes could simply reflect variations in vascular density across the layers and not neuronal populations. The present study provides direct evidence of the sensitivity of fMRI techniques to neuronal processes in different cortical layers by measuring stimulus-evoked *differential* layer-specific activations, i.e., changes in the distribution of the BOLD response across cortical depth under different stimulus conditions.

## Materials and Methods

Ethics statement: the experimental protocols for the experiments described below conformed to safety guidelines for MRI research and were approved by the Institutional Review Board at the University of Minnesota. Each subject participated in two or three scanning sessions, providing written informed consent after the nature of the experiments had been fully explained.

### Data acquisition

Functional MRI data were acquired using a 7 Tesla system on 3 subjects (2 male, ages 25–35), with 2 subjects returning for a second (control) scanning session with additional low-contrast stimuli. One of the 3 subjects returned again for a scanning session studying the laminar distribution of color-opponent responses and an additional subject (female, age 25) was also recruited for this experiment. The 7T system was equipped with a 90 cm bore, controlled by a Siemens (Erlangen, Germany) console and equipped with a Siemens head gradient set operating at up to 80 mT/m with a slew rate of 333 T/m/s. A half volume radio-frequency (RF) coil was used for transmission, and a small (6 cm) quadrature coil was used for reception [18].

Functional data were acquired with a 3D GRASE [19] pulse sequence: field of view was 2.2×17.9×0.48 cm^3^, matrix size was 32×256×8 (for a nominal resolution of 0.7×0.7×0.6 mm, the third dimension being sampled more finely to compensate for T_2_* blurring in the 2^nd^ phase-encode direction), echo train was ∼170 msec, TE/TR were 30/2000 msec. Data were acquired with 25% slice oversampling to eliminate confounding signal wrap-around in the 3D acquisition.

### Experiment design

Stimuli for the main experiment consisted of colored drawings of common objects on a white background [20]. The visual objects were masked by a stationary gray occluder and were therefore visible only through circles on a hexagonal grid (referred to as mask apertures, each with 2° diameter, separated by 0.7–1.0° of visual angle). (The occluder was not a physical occluder, but an inferred mask generated by setting pixel values to mean gray everywhere except in the specified circular apertures.) For the scrambled condition, the content of each circular aperture containing a part of an object was rotated by an angle drawn with equal probability from two uniform distributions: [60° 120°] or [−120° −60°]. A set of 188 images was divided into 2 groups: 94 were shown during the intact condition and 94 during the scrambled condition, to minimize the likelihood that subjects would recognize scrambled images by detecting familiar patches learned during presentation of intact images. The colored line drawings of objects were centered in the image, and because of variations in shape, image contrast was present in different regions of the visual field for different images. On average, however, the intact and scrambled objects provided the same image contrast to each visual field location (Figure S1). In some image regions, the intact and scrambled objects did differ in average orientation (e.g., near the vertical meridian, intact objects contained more horizontal orientations than their scrambled counterparts). Details are provided in Figure S1. This resulted in some low-level stimulus differences that may contribute to observed neural response differences between the stimulus conditions, a point that will be considered in the discussion. Visual stimuli were generated in Matlab and presented using the Psychtoolbox extensions [21], [22]. Subjects viewed the stimuli, which subtended ±7.6°, via a mirror mounted on the surface coil.

Intact and scrambled objects were presented during separate block-design scans, during which stimulus and rest alternated in 16s blocks, completing 10 ½ cycles for a total scan duration of 336 seconds (168 TRs). During the 16-second stimulus blocks, images were presented for 250 ms each (64 images per block, drawn at random from the set of 94 images). These stimulus blocks alternated with 16-second rest blocks. Throughout all scans, subjects were instructed to fixate on a red square at the center of the screen, pressing a button every time the square changed size. Attention was therefore not explicitly directed at the objects. As a control, to be sure that accidental differences in color or stimulus complexity between the two sets of images did not provide different strengths of input to V1 (e.g., T-junctions and curvature that would not be detected by the orientation analysis), scrambled versions of the objects from the “intact” group were shown as the first 2 scrambled scans for 2 of the 3 subjects. The average magnitude of the fMRI response to the two different types of scrambled object scan did not differ and were therefore grouped for subsequent analyses. Subjects completed between 10 and 14 scans, alternating between presentation of intact objects and scrambled objects.

### GLM

**Figure 1:**
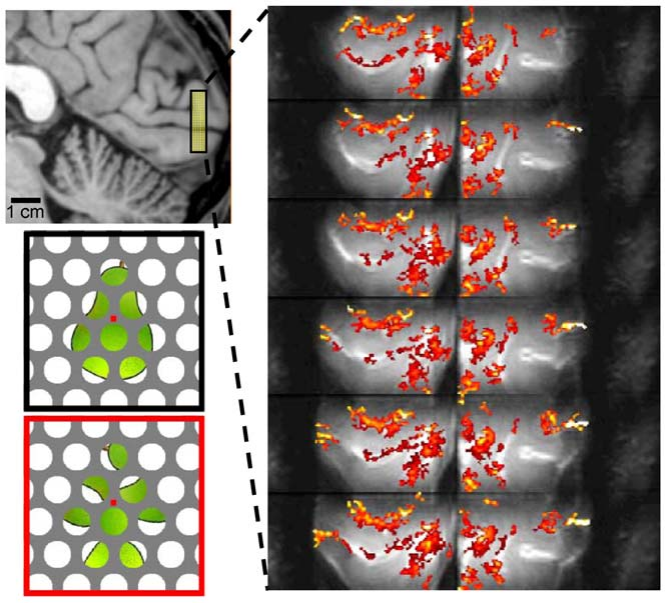
Volume coverage and activation of fMRI experiment with 0.7 mm resolution. Left: location of functional data is illustrated on a sagittal section. Right: activation maps from a single subject in response to both intact (bottom left, black outline) and scrambled objects (contrast is all-stimuli vs. blank, p<0.01, uncorrected, minimum cluster size 4 voxels).

The stimulus was modeled as a square wave (16 s on, 16 s off) convolved with a standard model of the hemodynamic impulse response function, generated by the function spm_hrf.m provided with SPM2 (http://www.fil.ion.ucl.ac.uk/spm/) using default parameters. After high-pass filtering the data (cut-off frequency: 4 cycles/scan, or 0.016 Hz), response amplitude was estimated by least-squares regression between the data and the stimulus model. Significance was estimated for each voxel by permutation analysis (randomizing the stimulus condition labels for each time point, while preserving the essential temporal correlation structure of the block design, and re-estimating the BOLD response modulation 1000 times) to estimate the probability (p-value) that the given coherence or modulation amplitude value would result from chance (Fig. 1, right panel).

### Cortical segmentation

Reference anatomical volumes were acquired with 0.7 mm isotropic resolution (proton-density-normalized MP-RAGE [23]). Cortical segmentation, along with gray matter (GM) and white matter (WM) surface definition, was performed on the reference anatomy using SurfRelax [24]. Cortical depth was quantified for each voxel as the *relative* distance from the WM surface (distance from WM divided by total cortical thickness at that location), which is more meaningful than *absolute* distance because of variation in cortical thickness throughout V1. Several distance metrics were tested: distances measured along normals to the WM surface, distances along lines connecting matched GM/WM surface vertices, and distances along the shortest line connecting each WM mesh vertex to the GM surface. All metrics produced comparable estimates of *relative* depth (Figure S2), so distances along connecting lines were used because this metric provided a label for the largest number of GM voxels.

### Registration of functional and anatomical data

Functional data were aligned to anatomical reference volumes using an intensity-based algorithm [25]. This alignment information was used to re-sample the following parameters from the anatomical reference space to the functional data space: V1 volume labels, eccentricity and polar angle information from previous retinotopic mapping sessions in each subject, and cortical depth estimates (Figures S3 and S4). Each functional voxel was thus classified as belonging to V1 or not and associated with a cortical depth and retinotopic location.

The definition of V1 in the functional data space was further restricted to contain only cortical regions where the tissue segmentation was high quality and alignment of functional and anatomical data was successful (while distortion was minimal in the 3D GRASE acquisition, it was not possible to optimize registration between functional and anatomical data throughout the entire slab). Two criteria were used for judging local GM/WM segmentation quality: local cortical thickness (measured by the length of lines connecting matched vertices on the GM and WM surface meshes) was less than 4 mm, and GM structure was evident under visual inspection (i.e., smooth progression was obvious from slice to slice). The criterion used to verify local registration between functional and anatomical data was that clusters of activated voxels should the follow GM contours. These conservative criteria limited analysis to regions where cortical curvature was relatively low and accurate alignment of functional and anatomical data could be visually verified, but still included between 1400 and 3300 voxels in the five experimental sessions analyzed.

### Laminar analysis

To generate laminar profiles, voxels were sorted according to relative depth (from 0 at the WM surface to 1 at the pial surface) and divided into five equally populated bins. After separating voxels by depth, the signals from all voxels in a bin were averaged before using linear regression to estimate the amplitude of BOLD response modulation (stimulus vs. rest) in each bin. This produced laminar profiles that sampled the cortical depth with a spacing of approximately 0.5 mm. The tortuosity of the cortex, with respect to the regular sampling of the fMRI voxels, results in continuous (not discrete) sampling of depths throughout the cortical ribbon and a spatial resolution that can be modeled as a Gaussian blurring kernel with full-width at half-maximum (FWHM) of 87% of the 0.7 mm isotropic voxel dimension [16]. Therefore, the response estimates in neighboring depth bins are not independent, but represent sampling of a smoothed laminar profile.

### Definition of clusters of activation

Next, within the subset of V1 that was (i) sampled by the 3D GRASE sequence, (ii) had a clean cortical segmentation, and (iii) demonstrated good alignment between functional and anatomical data, clusters of activated voxels were identified for further analysis. Because of the visual stimulus geometry, V1 was not uniformly stimulated. Instead, visual neurons representing the visual stimuli were expected to be clustered cortical volumes ranging from ∼8 mm diameter near the fovea (measured on the flattened cortical surface) to ∼3 mm diameter in the periphery, due to anisotropic visual stimulus representation as a consequence of cortical magnification. To facilitate identification of the cortical location of a cluster, data were smoothed (for the purpose of region-of-interest definition only) with a Gaussian kernel (σ = 1.4 mm). A cluster of activated voxels was defined in the smoothed data as a contiguous group of at least 100 voxels modulated significantly (p<0.001, uncorrected, in the smoothed data) by the presentation of visual stimuli (collapsing across all stimulus types). Regions of interest spanning the cortical depth and also including immediately adjacent white matter were drawn manually around all 11 clusters identified for the 5 scanning sessions.

Once a cluster of significantly modulated voxels was identified, the binning procedure described above was used to estimate the BOLD response to intact and scrambled objects in the WM and throughout the cortical depth. Clusters were excluded from further analysis if the estimated WM “activations” in response to intact and scrambled objects differed by more than 0.16% (two times the average standard error of estimated BOLD responses, averaged across all subjects). This instability in the baseline indicated poor data quality, either due to small cluster size or poor local alignment between functional and anatomical data. Four of the 11 identified clusters were excluded from further analysis by this criterion (Figure S5); all of the excluded ROIs had volumes less than 200 voxels. Therefore, in total 7 clusters met inclusion criterion for the calculation of differential laminar profiles.

### Experiment 2

As an additional control experiment, characterizing the laminar profiles of BOLD responses to feed-forward neural activity, full-field checkerboard stimuli were shown to two subjects. Checkerboards were either iso-luminant checkerboards defined by red and green squares at 2 cycles per degree (cpd) flickering at 1 Hz (targeting the parvocellular pathway) or rapidly flickering (15 Hz) checkerboards with luminance contrast at 0.3 cpd. Four 16-second blocks of each stimulus type were interleaved in scans repeated 6–8 times per subject, with 16 s rest between blocks and alternate scans starting with color-opponent stimuli. Separate localizer scans (high-contrast, black/white checkerboard, 16 s on/off block design; 3–4 repetitions per scanning session) identified V1 ROIs containing 1797 and 2329 voxels for the two subjects shown. Data were analyzed as in the main experiment, with a GLM estimating the fMRI response to each type of stimulus in the average signal from WM voxels and GM voxels separated into 5 bins through the cortical depth.

## Results

A T_2_-weighted 3D GRASE pulse sequence [19] with zoomed spatial resolution [26] was used to acquire fMRI data with 0.7 mm isotropic image resolution and 2 sec temporal resolution [27]. Due to the required very high spatial resolution, the sampled volume was small to make the image acquisition times feasible (Fig. 1; total fMRI volume was: 2.2×17.9×0.48 cm^3^). Data acquisition and analysis therefore focused on a subset of V1 (Figures S3 and S4). After intensity-based rigid-body alignment between the functional data and an anatomical reference volume, cortical depth information was sampled for each functional voxel in primary visual cortex.

**Figure 2:**
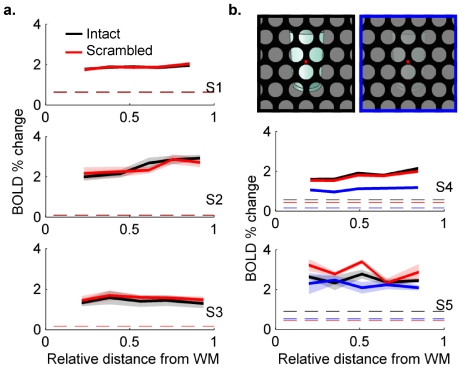
Laminar profiles of BOLD responses for significantly modulated voxels in V1. a.) Solid lines represent mean across repeated scans, shading indicates standard error across scans. Dashed lines represent average fMRI response in WM voxels. Significance threshold for defining activated voxels was adjusted for each subject to ensure a minimum of 300 voxels were included in the profile. S1: 1014/3287 voxels in V1 had p<0.001 (estimated by permutation analysis), 7 scans were averaged per condition; S2: 332/2036 V1 voxels with p<0.01, 5 scans per condition; S3: 325/1429 voxels in V1 with p<0.05, 5 scans per condition. b.) The control experiment included a set of scans in which the contrast of the intact objects reduced (image on top, right; blue laminar profiles). S4: 825/2953 of V1 voxels with p<0.001, 7 scans per condition; S5: 317/2126 of V1 voxels with p<0.05, 4 scans per condition.

First, laminar profiles were estimated for all V1 voxels that were significantly modulated by the presence of visual stimuli (collapsing across stimulus type). Differences were not observed in the shapes of the laminar profiles representing responses to intact vs. scrambled objects (Fig. 2), which were essentially flat through the cortical depth.

To verify that our method was sensitive to BOLD signal modulation at all depths, two of the subjects participated in a control experiment in which a third stimulus condition of reduced-contrast objects was presented, in addition to the original high-contrast intact and scrambled objects. (Experimental details were identical except objects were presented against a mean gray background to permit meaningful contrast manipulation.) This decrease in the strength of the input to V1 (as a result of reduced luminance contrast) should result in a decrease in the activity of neurons at all depths. The results (Fig. 2b) do indeed show decreases in fMRI signal throughout the cortical depth in response to decreased image contrast (blue lines). Laminar profiles of the fMRI responses to intact and scrambled objects were again not different. Considering the possibility that the valence of response modulation varies across V1 – some regions might respond more strongly to the presentation of intact objects while others might produce larger signal changes in response to scrambled objects – further analyses pursued local rather than global V1 response modulation.

**Figure 3:**
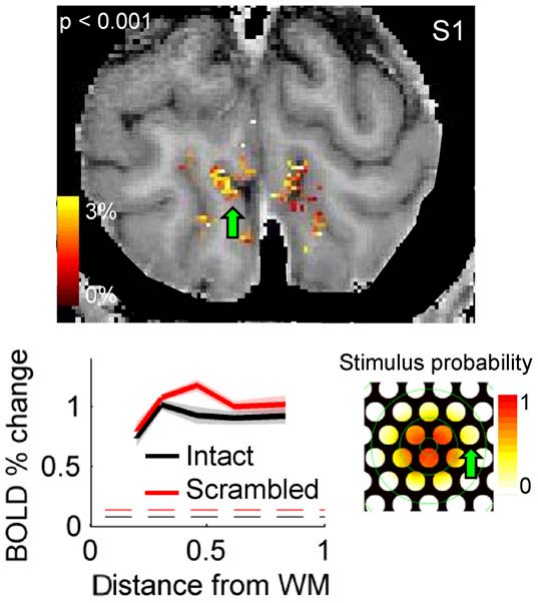
An example of a selected cluster of significantly modulated voxels. Voxels most strongly modulated by stimulus presentation (p<0.001) are shown overlaid on the T_1_-weighted anatomical reference, illustrating adherence of activation to GM. Map on lower right indicates probability that part of an intact or scrambled object occurred in each region of the visual field; image subtends ±7.6°. Green arrows indicate one region of interes and corresponding visual field location. Laminar profiles are shown with shading indicating standard error, 7 scans.

Because of the stimulus geometry, V1 response modulation is expected only in regions of cortex representing the isolated image features. Therefore, we proceeded to restrict the laminar analysis to clusters of strongly modulated voxels in each subject (example shown in Fig. 3). Comparison of the cortical location of the selected clusters of voxels against retinotopic data from previous scanning sessions verified that the clusters corresponded retinotopically to a single image location (one aperture in the occluding mask) in which features belonging to intact or scrambled objects appeared (see Figures S3 and S5 for details). To avoid biases from voxel selection, regions of interest were drawn around these clusters (without using statistical thresholds) to include all gray matter at the cortical location of the largest cluster of stimulated voxels in each subject's V1.

**Figure 4:**
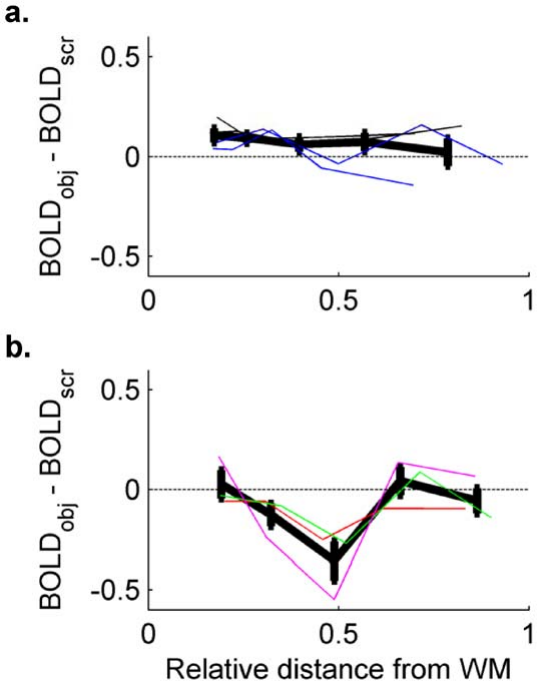
Differential laminar profiles of clusters sorted according to whether the average response to intact or scrambled objects was greater. a.) Regions of interest responding more strongly to intact objects were observed in 4 scanning sessions (colors of thin trace indicate different subjects). No main effect of depth is observed, although there is a statistically insignificant pattern of stronger responses to objects in deeper layers. b.) Regions of interest responding more strongly to objects were observed in 3 scanning sessions.

Seven clusters were identified in the regions of V1 covered in the five scanning sessions. While there was no main effect of depth in the response amplitude of all seven clusters (F_4,30_ = 0.84, p = 0.81), the fact that different clusters of voxels exhibited preferences for either intact or scrambled objects (Fig. 4, Figure S5) confounded interpretation. Regions of interest were therefore divided into two groups, based on whether the average response (across all depths) was greater to intact or scrambled objects. Differential laminar profiles (calculated by subtracting, at each depth, the response to scrambled objects from the response to intact objects) are shown in Fig. 4. In the four regions of interest that showed a preference for intact objects (Fig. 4a), there is no significant main effect of depth (F_4,15_ = 0.53, p = 0.71). In the regions responding more strongly to scrambled objects, however, an ANOVA shows a main effect of depth (F_4,10_ = 5.0, p = 0.018), with the largest differences occurring near the middle of the gray matter. (Figure S6 shows that the same pattern is evident when voxels are divided into 3, 4 or 6 depth bins.)

**Figure 5:**
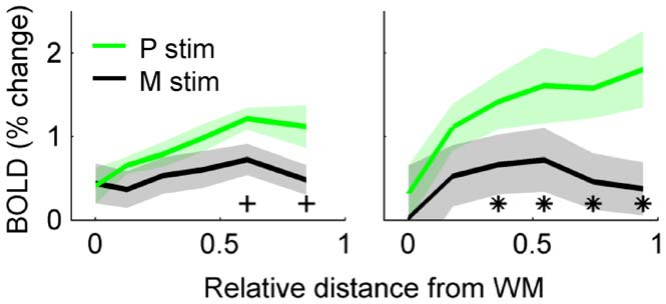
Visual responses to parvocellular-targeted stimuli are maximally differentiated from magnocellular-targeted stimuli in superficial gray matter. Full-field visual stimuli were targeted at the parvocellular pathway (P stim: 2 Hz contrast-reversing circular gratings composed of 1 cycle per degree iso-luminant red and green checks) and the magnocellular pathway (M stim: 15 Hz contrast-reversing gratings with 0.3 cycles per degree sinusoidal luminance modulation). Green lines indicate estimated BOLD response to P stimuli (shading indicates standard error across 6 or 8 scans in each subject, respectively); black lines indicate estimated BOLD response to M stimuli. Asterisks indicate significance at a given cortical depth (p<0.05, paired t-test, corrected for 10 comparisons in the 5 depth bins, 2 subjects); daggers indicate p<0.1, corrected.

The observed V1 laminar profile modulation in the object-detection experiment was strongest near the middle of the cortex depth, not in superficial or deep layers as might be predicted from *a priori* hypotheses about contextual modulation *via* either feed-back or local computations. This raises concerns about the sensitivity of the BOLD response to neural responses changes outside of the middle layers. Therefore, a second experiment was conducted using full-field contrast-reversing gratings designed to maximally stimulate magnocellular vs. parvocellular neural pathways (Fig. 5). The laminar distribution of color-opponent responses in V1 is well established: responses driven by high-spatial frequency, iso-luminant, color-opponent stimuli targeted at the parvocellular pathway should peak in more superficial layers because an estimated 60% of neurons in superficial layers are color opponent, as opposed to only 40% in deeper layers ([28] and references therein). As expected, color-opponent/low temporal frequency responses were larger than high temporal frequency/luminance-driven responses in superficial V1 gray matter in two subjects (Fig. 5). This confirms that layer-specific fMRI analyses of T_2_-weighted data can reflect differential signals outside the middle layers.

## Discussion

To our knowledge, these are the first neuroimaging observations of laminar profiles reflecting sensitivity to a change in the distribution of neural population activity through the cortical depth in response to any stimulus modulation or perceptual state. Previous concerns about the interpretation of layer-specific fMRI were: (i) the dominance of signal from large veins on the pial surface, (ii) the elaboration of the cortical vasculature in the middle of the gray matter depths, and (iii) the possibility that functional signals, even with acquisitions biased toward the parenchymal signal, as in this study, will be dominated by penetrating intracortical venuoles [29] that pool blood signal across layers, rendering layer-specific fMRI impossible. Each of these concerns represents a different aspect of the vascular architecture that might limit the ability of fMRI to reflect modulations of neural responses at different depths, but the present study demonstrates that – in spite of these baseline biases in the fMRI signal – sensitivity to neural modulation at different depths is possible.

The present study was accomplished with a T_2_-weighted pulse sequence (3D GRASE) in order to improve the homogeneity of the functional resolution by minimizing sensitivity to pial signal throughout the cortical thickness. Degradation of the spatial specificity of the GE BOLD signal by pial veins in superficial layers has been established [15]. While one can identify and attempt to remove voxels contaminated by pial vein signal [14], parenchymal and pial signal are unpredictably confounded in these superficial voxels, so the result of this approach (cleaning up GE BOLD data) is (i) a loss of sensitivity to fMRI signal arising from neurons in superficial layers, and (ii) a potentially compromised middle layer response, in cases where extravascular signal from large-diameter veins spreads >1 mm. While the sensitivity of the 3D GRASE signal to contrast arising in different parenchymal venous compartments is not fully characterized, and the acquisition strategy is potentially vulnerable to degradation of spatial resolution in the through-slice direction due to the 3D nature of the acquisition, the T_2_-weighting of the 3D GRASE acquisition is certain to improve the homogeneity of the resolution by minimizing specificity problems introduced by the pial vein signal [30].

The second concern mentioned above is that strong fMRI responses in the middle of the GM represents only the local elaboration of the vasculature. This concern is based on previously published single-condition laminar profiles that show a stronger GE BOLD response in middle layers, which could be consistent with either greater synaptic density or greater vascular elaboration, and a peak in superficial depths, attributed to the presence of pial veins [13], [14], [16]. (At the moment, there are conflicting reports on the presence of a significant increase in the functional response in the middle of the cortex, but these may be in part due to the different stimuli used in different studies – features such as temporal frequency, spatial frequency and chromatic content should modify laminar profiles if they are sensitive to the distribution of local neural computations). The laminar profiles generated for all significantly modulated V1 voxels (Fig. 2) demonstrate the profile expected from a T_2_-weighted sequence, which in some subjects shows a *slight* increase in the middle of the cortical thickness but in all subjects lacks the peak in superficial GM caused by large pial veins. The small ROI-based laminar profiles generated in this study (Fig. 3, Figure S5) do not always follow this pattern, possibly due to their limited spatial extent (230–360 voxels, for an average of 60 voxels per bin), which makes them highly susceptible to small errors in alignment or segmentation. Fortunately, these potential errors have minimal impact on conclusions based on differential laminar profiles, as any errors would affect both stimulus conditions equally.

The third concern about the contribution of vascular geometry to depth-dependent fMRI responses is the vertical organization of vascular architecture. Recent studies support this concern: measurement of the onset time of the BOLD response as a function of cortical depth [31], [32] shows a regular progression of the hemodynamic response from deep layers to superficial layers, and from the trunk of arterioles into the capillary bed. The systematic change in timing along the length of penetrating intracortical arterioles questions whether there is independent control of the hemodynamic response at different cortical depths. Analysis of the depth dependence of response timing in our data (Figure S7) shows the same pattern: responses in deep layers precede responses in superficial layers by 100 ms or more. Therefore, the current data suggest that, like the laminar bias in the baseline amplitude profile, there is a laminar bias in the baseline onset times for the fMRI response that does not preclude detection of stimulus-specific information at different cortical depths.

The data collected using fast, coarse, luminance-modulated stimuli (magnocellular-targeted) versus slow, fine, color-opponent stimuli (targeted at the parvocellular pathway) revealed stronger modulation of the laminar profiles in superficial layers (Fig. 5), away from the vascular elaboration in the middle of the cortex. These data provide compelling evidence for the sensitivity of BOLD to different neural computations at different depths. It is possible that the large color-opponent response in superficial layers is due, at least in part, to the extra vascularization in superficial cytochrome oxidase blobs [33]. However, this line of argument also suggests that the main experiment – and any experiment using colorful, naturalistic stimuli –benefits from strong fMRI sensitivity to neural response modulation in superficial layers, as well as middle layers. Therefore, these data again show that, while vascular geometry may bias the baseline laminar profile in middle and/or superficial layers, fMRI remains sensitive to different neural computations at different depths.

Because subjects were engaged in a fixation task during the presentation of intact and scrambled objects during the main study, attention was not directed at the visual objects and subjects provided only post-scanning reports about their awareness of the differences between the scan types. This design was selected because not all subjects were experienced psychophysical observers, and fixation stability was paramount. More detailed behavioral reports may have helped to explain the variability between scanning sessions observed in differential laminar profiles (Fig. 4): when regions of interest were sorted by whether the overall response amplitude was greater for intact or scrambled objects, the ROIs preferring intact objects came from two scanning sessions, while the ROIs preferring scrambled objects came from three other scanning sessions. Better cognitive control over, or more complete reports describing, subjects' perceptual state may have reduced and/or explained this variance.

The most reliable pattern of results that emerged from the main experiment was in ROIs showing stronger overall fMRI responses to scrambled objects. This stronger response to scrambled objects is consistent with previous reports of reduced BOLD response during perception of coordinated, rather than disjoint or unconnected, image features [5]. In these regions, the larger response to scrambled objects was limited to middle layers. With the present resolution, we cannot separate input responses in Layer 4C from local computations in Layer 4B. Nor can the unidimensional experiment design determine whether the measured laminar profiles are the result of one or many neuromodulatory processes. However, a major goal of high-resolution, layer-specific imaging is to determine whether these observed modulations of V1 responses are effected by local, feed-forward or feed-back neural mechanisms. While this experiment does not provide conclusive evidence for or against any specific neural mechanism, it is worth discussing the neural mechanisms consistent with this pattern of results that are useful to consider in the design of future experiments.

First, it is possible that re-entrant cortico-thalamo-cortical circuits [34] contribute to the observed V1 modulation. The increased response to scrambled objects in middle layers is consistent with stronger thalamo-cortical activity in the scrambled condition. However, increased input to middle layers should simultaneously produce stronger responses in deep and superficial layers (as in the control experiment), unless additional neural processes are differentially affecting neural responses in Layers 2/3 and 5/6. Cortico-cortical feed-back enhancement of V1 responses in deep and superficial layers [35] during perception of intact objects is one mechanism that, in combination with increased V1 input during presentation of scrambled objects, could produce the pattern of results we observe. Therefore, while our data can rule out a simple increase in input to V1 during presentation of scrambled objects, more studies are clearly required to delineate and disambiguate possible neuronal mechanisms for modulation of primary visual cortex during object recognition tasks.

Second, as mentioned above, cortico-cortical feedback is a possible source of the observed laminar differences. Our *a priori* hypothesis was that feedback to superficial and deep layers of V1 would enhance neural activity outside of Layer 4 upon recognition of intact objects (consistent with [4] rather than [5]). This would result in a laminar profile that was relatively smaller in the middle for intact objects. In part, this is what we observed, but the relative ordering of the BOLD responses in the middle layers (scrambled > intact) is not consistent with the *a priori* hypothesis. Our data do not rule out the possibility of feature-selective cortico-cortical enhancement of intact object representations, but they indicate that, if such a mechanism is at work, it is not the only mechanism affecting the V1 neural activity.

Finally, it is possible that the observed differences in neural response profile are due at least in part to local V1 computations (i.e., cortico-cortical feed-back mechanisms are not necessary to explain our pattern of results). Even V1 neurons with spatially restricted receptive fields could detect differences in feature colinearity in the two conditions, since the gaps between apertures were less than 1 degree of visual angle. There is therefore no need to invoke high-level visual or cognitive effects to explain the sensitivity of V1 to scene organization observed in this experiment. Because laminar profile differences were observed in clusters corresponding to regions within apertures and not between apertures, the spatial distribution of the differences observed in the laminar profiles does not strongly support a V1-intrinsic source (i.e., responses in neurons with receptive fields spanning two apertures). However, long range horizontal connections, such as those that might response to the increased colinearity in the intact condition, are distributed preferentially in superficial layers and could affect the laminar profile in response to intact objects in ways that are difficult to distinguish from cortico-cortical feed-back targeted at superficial layers. Similarly, scrambling by rotation was used to maintain the identity of low-level features while removing the large-scale coordination of features that results in scene identity. However, orientation content differed systematically (and necessarily) across the images as objects were scrambled (Figure S1). There was no systematic match between differential laminar profile and image location, so the variability in differential laminar profiles cannot be explained by the variability in orientation content (Figures S1 and S5). However, this divergence in orientation distribution away from naturalistic values [36] is another example of a low-level neural mechanism that could cause different feed-forward neural response patterns when local features are rotated.

We have presented four possible neural mechanisms – sensitivity to local image statistics, re-entrant processing, cortico-cortical feed-back, and local computations – that might separately or in combination create the pattern of laminar profile modulation that we measured. In particular, we have noted that local computations and neural feed-back mechanisms cannot be distinguished based solely on measured laminar profiles. This points to an important caveat for the interpretation of laminar profiles – the fact that local and feed-back mechanisms often target the same populations of neurons means that a single layer-specific fMRI experiment will never be sufficient, on its own, to distinguish feed-forward and feed-back neural mechanisms. Furthermore, intra-laminar connectivity in each cortical area/column, as well as the fact that multiple neural mechanisms are often simultaneously at work, significantly complicates interpretation of laminar profiles. Nevertheless, the complexity of the interpretation of laminar fMRI profiles does not mitigate our excitement at this demonstration the fMRI can be sensitive to different neural responses at different depths.

A further motivation for this study is that, while many human fMRI studies have reported strong increases or decreases in the V1 BOLD response during perceptual changes [2], [4], [5], [37], electrophysiology studies have for the most part observed much more subtle differences [2]. The use of very high-resolution T_2_-weighted fMRI to localize changes to specific cortical layers could help specify which sub-populations of neurons are driving the observed BOLD response changes, providing data on a scale better-matched to the local field potentials detected by invasive electrophysiology. Thus, the high spatial specificity fMRI responses demonstrated in this study may help resolve discrepancies between fMRI and electrophysiological measurements by bridging the large-scale cortical coverage available through fMRI and the detailed measurements of neural population activity available through invasive electrophysiology.

One notable challenge in detecting layer-specific activation profiles is achieving sub-millimeter resolution in human subjects. The voxel dimensions for this experiment were 0.7 mm (isotropic); as noted in the methods, curvature in the cortex means that cubic voxels sample cortical activation with a point-spread function characterized by a Gaussian kernel with 0.6 mm FWHM. Additional blurring, however, results from T_2_*-blurring (minimized by the fast image readout using head gradients) and image distortion, so the true resolution of the experiment is likely best characterized by a Gaussian kernel with a FWHM of approximately 1 mm. This does not mean that information on the ∼0.5 mm spatial scale necessary for laminar analyses is inaccessible; it only means that the contrast between layers is attenuated by blurring caused by sampling errors and subject motion. Further decreasing the voxel dimensions in future experiments – which will require RF coils with increased sensitivity and the incorporation of parallel imaging techniques in the 3D GRASE acquisition – will mitigate this problem.

Advances in fMRI imaging technology currently being pursued (i.e., surface array coils, parallel imaging, etc.) will result in improvements in sensitivity, efficiency, and volume coverage of high resolution acquisitions, which will in turn allow for an expanded subject pool and more powerful event-related designs, or experiments measuring laminar profiles in multiple cortical areas, to gather more precise interpretation of the correlation between cognitive state and observed mechanisms. As they stand, however, these data provide compelling evidence that fMRI investigations of neuronal computations at different depths will yield new information about how the brain encodes behavioral information.

## Supporting Information

Figure S1 **Analysis of RMS contrast and orientation content of intact and scrambled objects.** a.) For each aperture fully contained in the ±7.6° subtense of the image, the percentage of trials (64 0.25 sec trials per block, 10 blocks per scan, 5–7 scans for each stimulus type per scanning session) on which part of an object appeared in an aperture was calculated (top). Dashed line indicates stimulus present 25% of the time. Apertures are numbered from left to right, top to bottom. Bottom: average RMS contrast during presentations of intact (black) and scrambled (red) objects. b.) Orientation content was estimated for 45×45 pixel patches centered on the 12 apertures (circled in green, top) that contained image parts on at least 25% of the trials (vignetted by a Gaussian to avoid edge artifacts, σ = 8 pixels, middle panel) by averaging the power in four orientation bands in the Fourier domain (bottom). The 12 plots at far right indicate power in the four different orientation bands for the 12 selected apertures; plots with a gray background indicate regions of visual space where orientation content differed significantly between intact and scrambled objects. Horizontal orientations are disproportionately present in patches on the vertical meridia for intact objects. Because patches were scrambled by an average rotation of 90°, scrambled objects show disproportionate representation of vertical orientations on the vertical meridia and horizontal orientations on the horizontal meridia.
(TIF)

Figure S2 **Methods for calculating gray matter depth.** a.) GM (yellow) and WM (blue) surfaces for each hemisphere were defined as pairs of triangulated meshes with the same numbers of faces (using SurfRelax). Surfaces are visualized on the reference anatomy from which they were defined: a 0.7 mm (isotropic) MP-RAGE volume in which signal intensity has been normalized (while preserving T1 contrast) by dividing the MP-RAGE volume by a proton-density weighted volume acquired in the same (interleaved) acquisition. b.) Voxels were assigned a depth (color overlay, scale bar in mm) by calculating the distance from the WM surface to the center of the voxel along lines traversing the voxel and connecting matched GM and WM vertices. Each voxel is assigned a depth that is the average of all WM/GM lines traversing the voxels. This method of measuring the GM thickness is referred to as the “matched faces” method. c.) To account for normal variation in cortical depth within and between subjects, relative distance from the WM, rather than absolute distance shown in (b.), was used for generating laminar profiles. Relative distance was calculated by dividing each voxel's distance from the WM by the local cortical thickness (average length of the WM/GM connecting lines traversing the voxel). Alternate distance metrics, such as the distances along lines normal to the WM surface or along lines connecting WM faces to the nearest GM face, were also tested. These metrics provide slightly different estimates for the *absolute* cortical depth in regions of high curvature (not shown), but identical estimates for the *relative* depth of each GM voxel in V1. Each black dot represents one voxel and is plotted with the abscissa value indicating the distance from the WM along lines connecting *matched faces*, and the ordinate value indicating the distance from the WM along *normals* extended to the GM surface. The red line indicates where data would lie if the two metrics provided identical estimates for each voxel's distance from the WM.
(TIF)

Figure S3 **Anatomical and functional overlay data for all subjects/scanning sessions.** Left: parasagittal T_1_-weighted image (acquired with the same surface coil as the functional data, normalized by division by proton-density image), with hot colormap overlay indicating position of functional imaging slab. Functional data were acquired over a volume covering 4.8 mm in the anterior/posterior direction, 22.7 mm in the superior/inferior direction, and 179.2 mm in the right/left direction. Within- and between-scan registration of functional data was accomplished using the 3dvolreg tool from AFNI. Subjects completed 5–7 scans of each type; 1–2 scans were discarded because of motion (displacement of the center of mass in a single scan, relative to the center of mass of all scans) greater than 1 mm detected by motion compensation. Motion-compensated data were aligned to anatomical reference data (also with 0.7 mm isotropic resolution) using intensity-based rigid-body registration (after inversion of image contrast) implemented in Matlab (using mrAlign: http://gru.brain.riken.jp/doku.php/mrTools/overview). Anatomical and retinotopic mapping data were then sampled from the anatomical reference space to assign each functional voxel in the motion-corrected dataset a GM depth and retinotopic location. Right, from top to bottom for each subject: cortical depth derived from lines connecting corresponding GM and WM surfaces (matched faces); local GM thickness as measured by the average length of GM/WM connecting lines traversing each voxel; eccentricity of visual field representation estimated from separate retinotopic mapping session (1–3°, 3–6° and 6–9°); polar angle of visual field representation. Note the very limited coverage of V1 due to strong hemisphere asymmetry in S3.
(TIF)

Figure S4 **Visual field locations of stimuli and retinotopic coverage of V1 in the functional data slab.** The square image containing the visual stimuli subtended ±7.6° of visual angle in the horizontal and vertical dimensions. Upper left panel: the probability that part of an object (either intact or scrambled; both categories had equal probability of occurrence in each aperture) was present in a given aperture on a given trial (red indicates p = 1; white indicates regions where object features were never present). The green circles marking 1°, 3°, 6° and 9° eccentricity overlaid on this map of stimulus intensity are the same as on the 5 polar grid plots that indicate retinotopic coverage in the small 3D GRASE slab prescribed for the functional study. To display information about retinotopic locations of activated clusters, the visual field is divided into 3 concentric rings (1–3°, 3–6° and 6–9° eccentricity, boundaries indicated by solid lines) and 8 wedges (not indicated, each occupying 45° of polar angle). Every GM voxel in V1 is assigned to one eccentricity bin and one polar angle bin based on retinotopic information from a previous scanning session transformed into the same reference anatomical space as the functional data from this study. The size of the black circle indicates the number of voxels assigned to a given polar angle or eccentricity bin in each subject. While this coarse division of the visual field is used here for displaying visual field coverage in each subject, retinotopic location of clusters of stimulated voxels in each subject were identified by inspection on flattened cortical surfaces and comparison against the fine-grained retinotopic data generated from separate rotating wedge/expanding ring (traveling wave) retinotopic mapping experiments conducted in separate scanning sessions.
(TIF)

Figure S5 **Laminar profiles for all ROIs defined in all subjects.** Each row is a different subject, each column is a different ROI. Dark gray backgrounds indicate ROIs excluded due to small size (producing unreliable estimates of BOLD response due to averaging fewer than 40 voxels per bin). Light gray background and/or asterisk and dagger indicate ROIs in a region of the visual field for which visual stimuli were present less than 25% of the time (therefore not included in further analyses). Numbers in upper left of each panel indicate index of ROI, as shown in inset at right (on which yellow numbers indicate ROIs without orientation bias and red numbers indicate ROIs with orientation bias). Color of plot axes indicates whether average response in ROI was stronger for scrambled (red) or intact (black) objects.
(TIF)

Figure S6 **Dependence of laminar profiles on number of bins.** Analysis for the main paper used 5 bins (3^rd^ column). Plots in top row are for ROIs showing a larger average response for intact objects; bottom row shows ROIs with stronger average responses to scrambled objects. Text in upper left of each panel indicates F-statistic and p-value from an ANOVA considering the main effect of depth.
(TIF)

Figure S7 **Dependence of onset timing on cortical depth.** All scans in a scanning session were averaged together, voxels were divided into five equally-populated depth bins, then for each depth all voxels in a bin were averaged. BOLD response onset was characterized by the phase of the stimulus-related Fourier component at the block-alternation frequency (10 cycles/scan). Stimuli alternated with a 32-second cycle, so the sinusoid phase (a value between 0 and 2π) was scaled to cover the range 0–32 sec. The mean latency was subtracted from the latency profile for each subject (thin black lines) before averaging latencies across subjects. An ANOVA shows a main effect of depth on latency (F_4,20_ = 4.61, p = 0.0084), consistent with previous reports of shorter onset latencies at the distal extent of penetrating intracortical arterioles.
(TIF)
