# Supplementary figures and images for: Layer-Specific fMRI Reflects Different Neuronal Computations at Different Depths in Human V1
Source: PLoS One. 2012 Mar 20;7(3):e32536. doi: 10.1371/journal.pone.0032536 (PMC3308958; doi:10.1371/journal.pone.0032536)

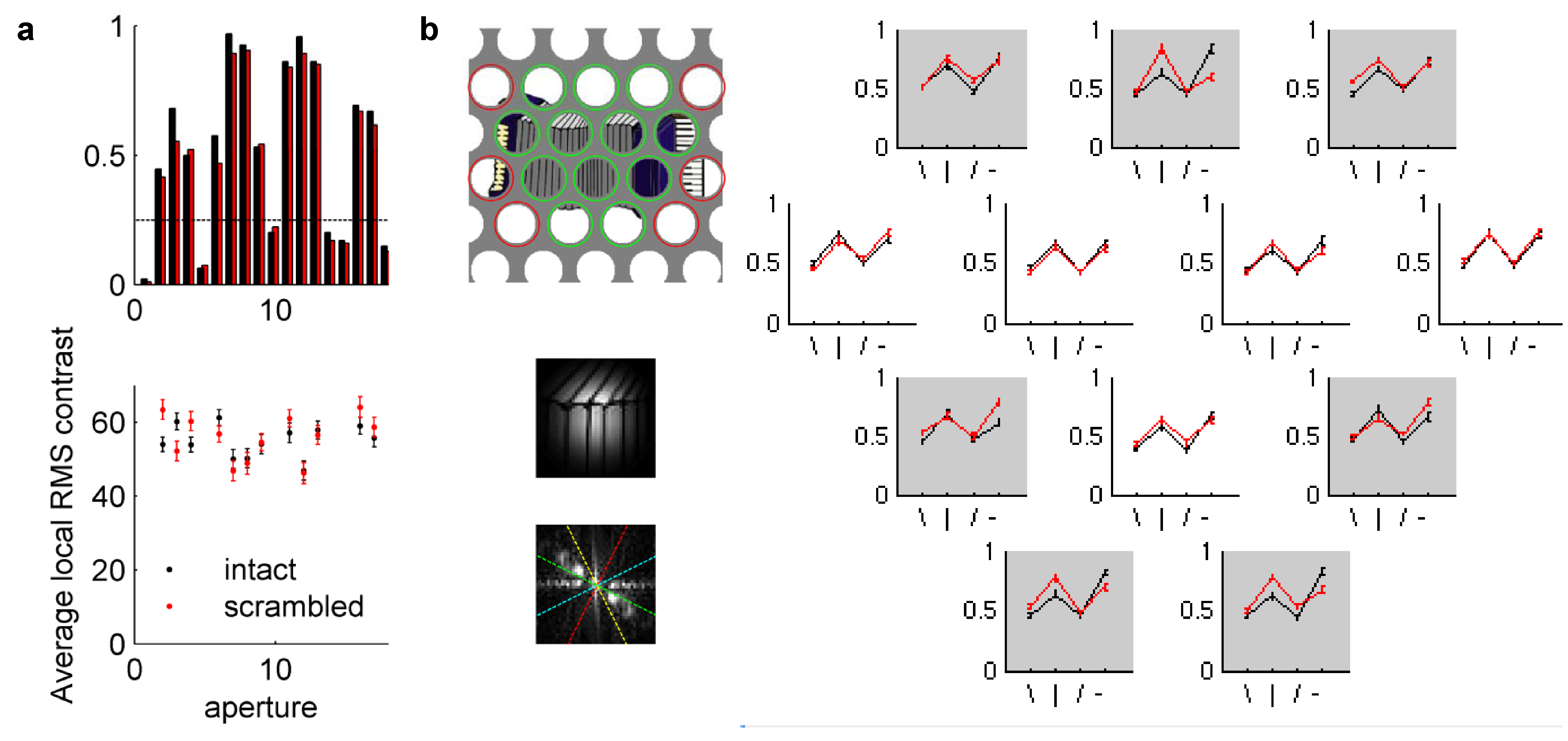

Supplement: Figure S1 — Analysis of RMS contrast and orientation content of intact and scrambled objects. a.) For each aperture fully contained in the ±7.6° subtense of the image, the percentage of trials (64 0.25 sec trials per block, 10 blocks per scan, 5–7 scans for each stimulus type per scanning session) on which part of an object appeared in an aperture was calculated (top). Dashed line indicates stimulus present 25% of the time. Apertures are numbered from left to right, top to bottom. Bottom: average RMS contrast during presentations of intact (black) and scrambled (red) objects. b.) Orientation content was estimated for 45×45 pixel patches centered on the 12 apertures (circled in green, top) that contained image parts on at least 25% of the trials (vignetted by a Gaussian to avoid edge artifacts, σ = 8 pixels, middle panel) by averaging the power in four orientation bands in the Fourier domain (bottom). The 12 plots at far right indicate power in the four different orientation bands for the 12 selected apertures; plots with a gray background indicate regions of visual space where orientation content differed significantly between intact and scrambled objects. Horizontal orientations are disproportionately present in patches on the vertical meridia for intact objects. Because patches were scrambled by an average rotation of 90°, scrambled objects show disproportionate representation of vertical orientations on the vertical meridia and horizontal orientations on the horizontal meridia. (TIF) [file pone.0032536.s001.tif]

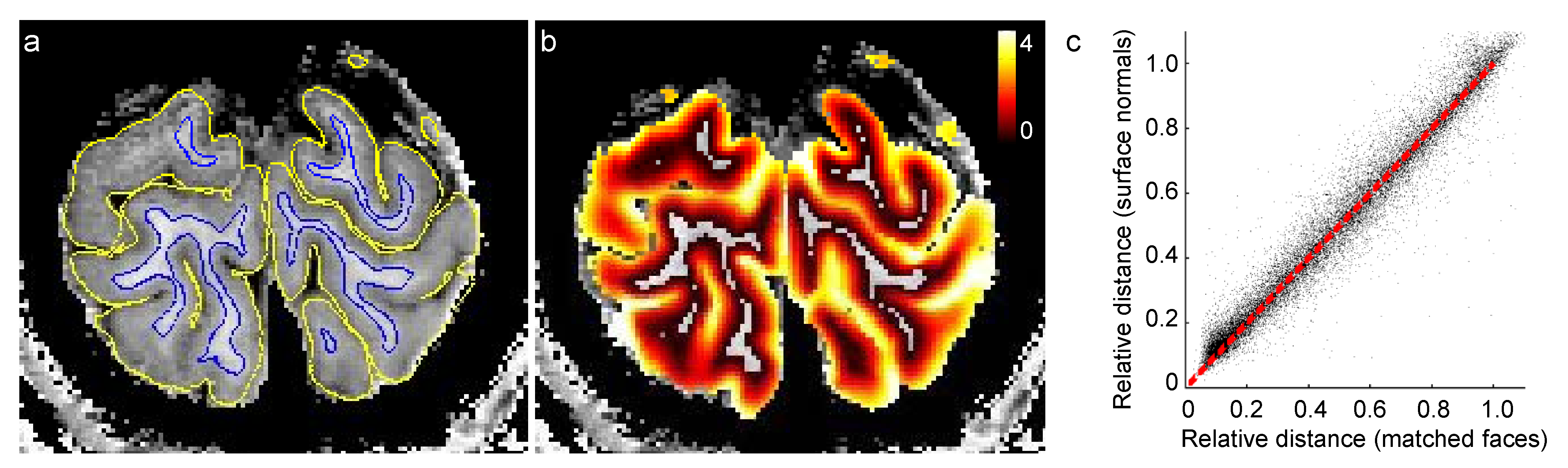

Supplement: Figure S2 — Methods for calculating gray matter depth. a.) GM (yellow) and WM (blue) surfaces for each hemisphere were defined as pairs of triangulated meshes with the same numbers of faces (using SurfRelax). Surfaces are visualized on the reference anatomy from which they were defined: a 0.7 mm (isotropic) MP-RAGE volume in which signal intensity has been normalized (while preserving T1 contrast) by dividing the MP-RAGE volume by a proton-density weighted volume acquired in the same (interleaved) acquisition. b.) Voxels were assigned a depth (color overlay, scale bar in mm) by calculating the distance from the WM surface to the center of the voxel along lines traversing the voxel and connecting matched GM and WM vertices. Each voxel is assigned a depth that is the average of all WM/GM lines traversing the voxels. This method of measuring the GM thickness is referred to as the “matched faces” method. c.) To account for normal variation in cortical depth within and between subjects, relative distance from the WM, rather than absolute distance shown in (b.), was used for generating laminar profiles. Relative distance was calculated by dividing each voxel's distance from the WM by the local cortical thickness (average length of the WM/GM connecting lines traversing the voxel). Alternate distance metrics, such as the distances along lines normal to the WM surface or along lines connecting WM faces to the nearest GM face, were also tested. These metrics provide slightly different estimates for the absolute cortical depth in regions of high curvature (not shown), but identical estimates for the relative depth of each GM voxel in V1. Each black dot represents one voxel and is plotted with the abscissa value indicating the distance from the WM along lines connecting matched faces, and the ordinate value indicating the distance from the WM along normals extended to the GM surface. The red line indicates where data would lie if the two metrics provided identical estimates for each voxel' [file pone.0032536.s002.tif]

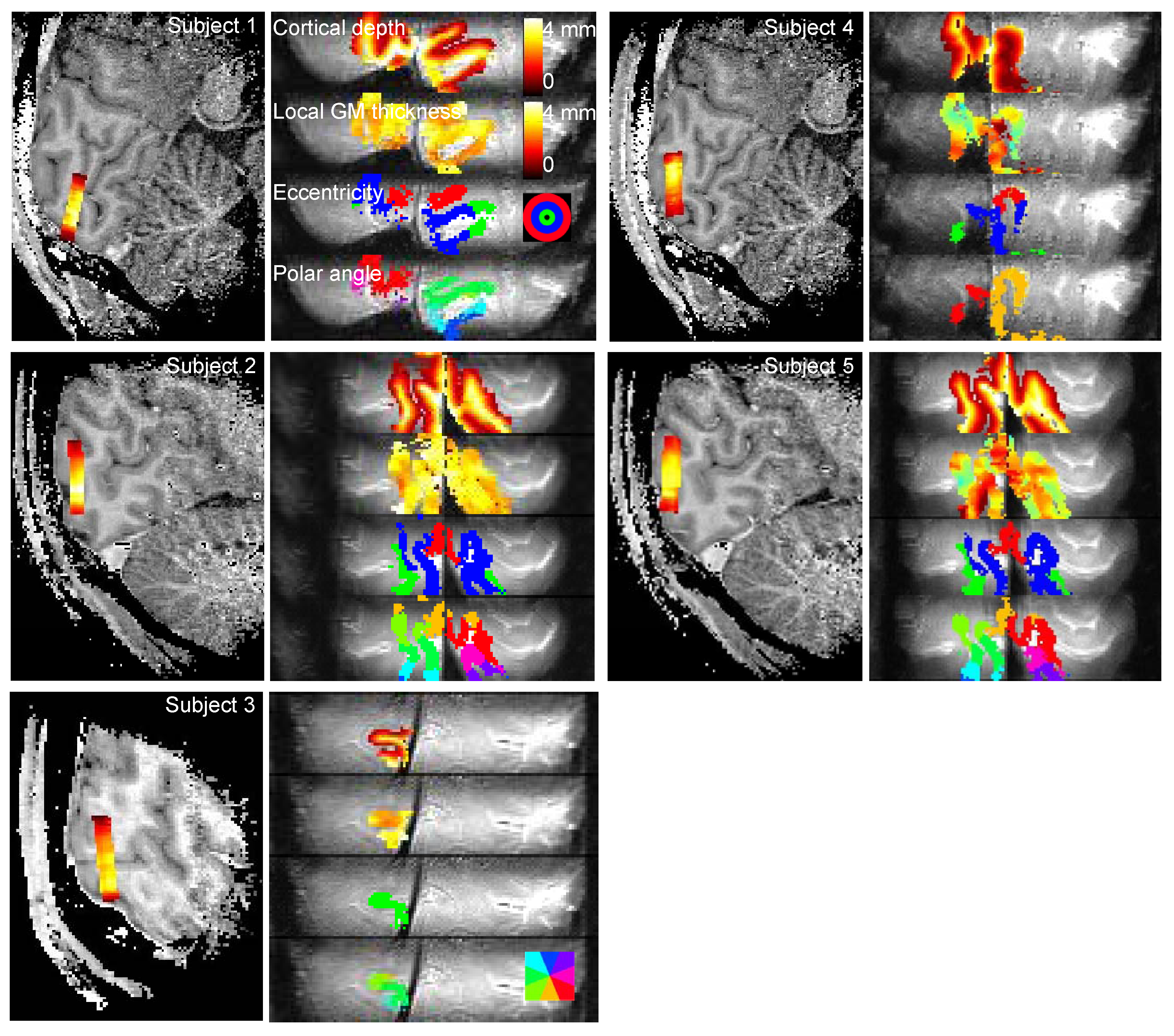

Supplement: Figure S3 — Anatomical and functional overlay data for all subjects/scanning sessions. Left: parasagittal T1-weighted image (acquired with the same surface coil as the functional data, normalized by division by proton-density image), with hot colormap overlay indicating position of functional imaging slab. Functional data were acquired over a volume covering 4.8 mm in the anterior/posterior direction, 22.7 mm in the superior/inferior direction, and 179.2 mm in the right/left direction. Within- and between-scan registration of functional data was accomplished using the 3dvolreg tool from AFNI. Subjects completed 5–7 scans of each type; 1–2 scans were discarded because of motion (displacement of the center of mass in a single scan, relative to the center of mass of all scans) greater than 1 mm detected by motion compensation. Motion-compensated data were aligned to anatomical reference data (also with 0.7 mm isotropic resolution) using intensity-based rigid-body registration (after inversion of image contrast) implemented in Matlab (using mrAlign: http://gru.brain.riken.jp/doku.php/mrTools/overview). Anatomical and retinotopic mapping data were then sampled from the anatomical reference space to assign each functional voxel in the motion-corrected dataset a GM depth and retinotopic location. Right, from top to bottom for each subject: cortical depth derived from lines connecting corresponding GM and WM surfaces (matched faces); local GM thickness as measured by the average length of GM/WM connecting lines traversing each voxel; eccentricity of visual field representation estimated from separate retinotopic mapping session (1–3°, 3–6° and 6–9°); polar angle of visual field representation. Note the very limited coverage of V1 due to strong hemisphere asymmetry in S3. (TIF) [file pone.0032536.s003.tif]

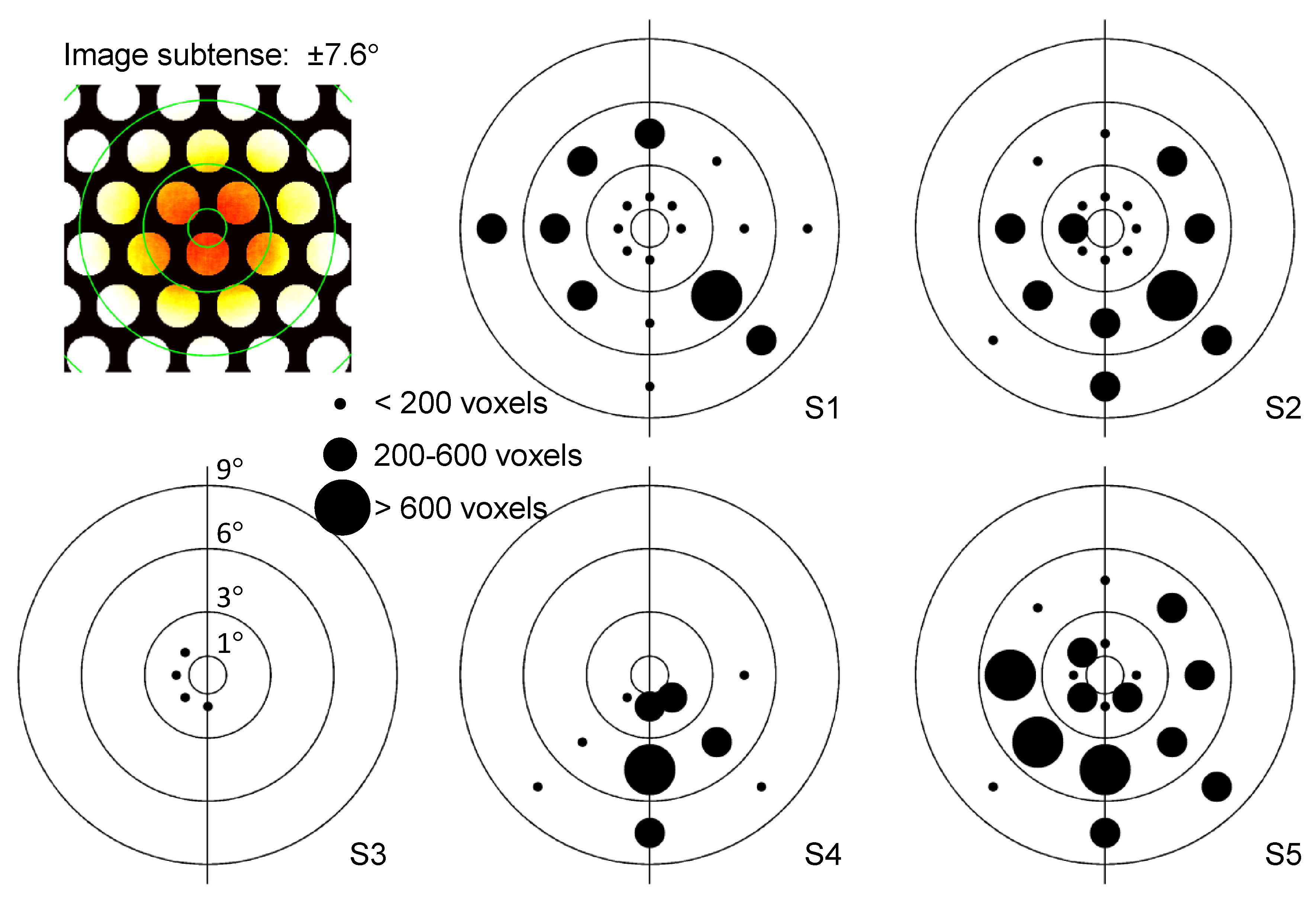

Supplement: Figure S4 — Visual field locations of stimuli and retinotopic coverage of V1 in the functional data slab. The square image containing the visual stimuli subtended ±7.6° of visual angle in the horizontal and vertical dimensions. Upper left panel: the probability that part of an object (either intact or scrambled; both categories had equal probability of occurrence in each aperture) was present in a given aperture on a given trial (red indicates p = 1; white indicates regions where object features were never present). The green circles marking 1°, 3°, 6° and 9° eccentricity overlaid on this map of stimulus intensity are the same as on the 5 polar grid plots that indicate retinotopic coverage in the small 3D GRASE slab prescribed for the functional study. To display information about retinotopic locations of activated clusters, the visual field is divided into 3 concentric rings (1–3°, 3–6° and 6–9° eccentricity, boundaries indicated by solid lines) and 8 wedges (not indicated, each occupying 45° of polar angle). Every GM voxel in V1 is assigned to one eccentricity bin and one polar angle bin based on retinotopic information from a previous scanning session transformed into the same reference anatomical space as the functional data from this study. The size of the black circle indicates the number of voxels assigned to a given polar angle or eccentricity bin in each subject. While this coarse division of the visual field is used here for displaying visual field coverage in each subject, retinotopic location of clusters of stimulated voxels in each subject were identified by inspection on flattened cortical surfaces and comparison against the fine-grained retinotopic data generated from separate rotating wedge/expanding ring (traveling wave) retinotopic mapping experiments conducted in separate scanning sessions. (TIF) [file pone.0032536.s004.tif]

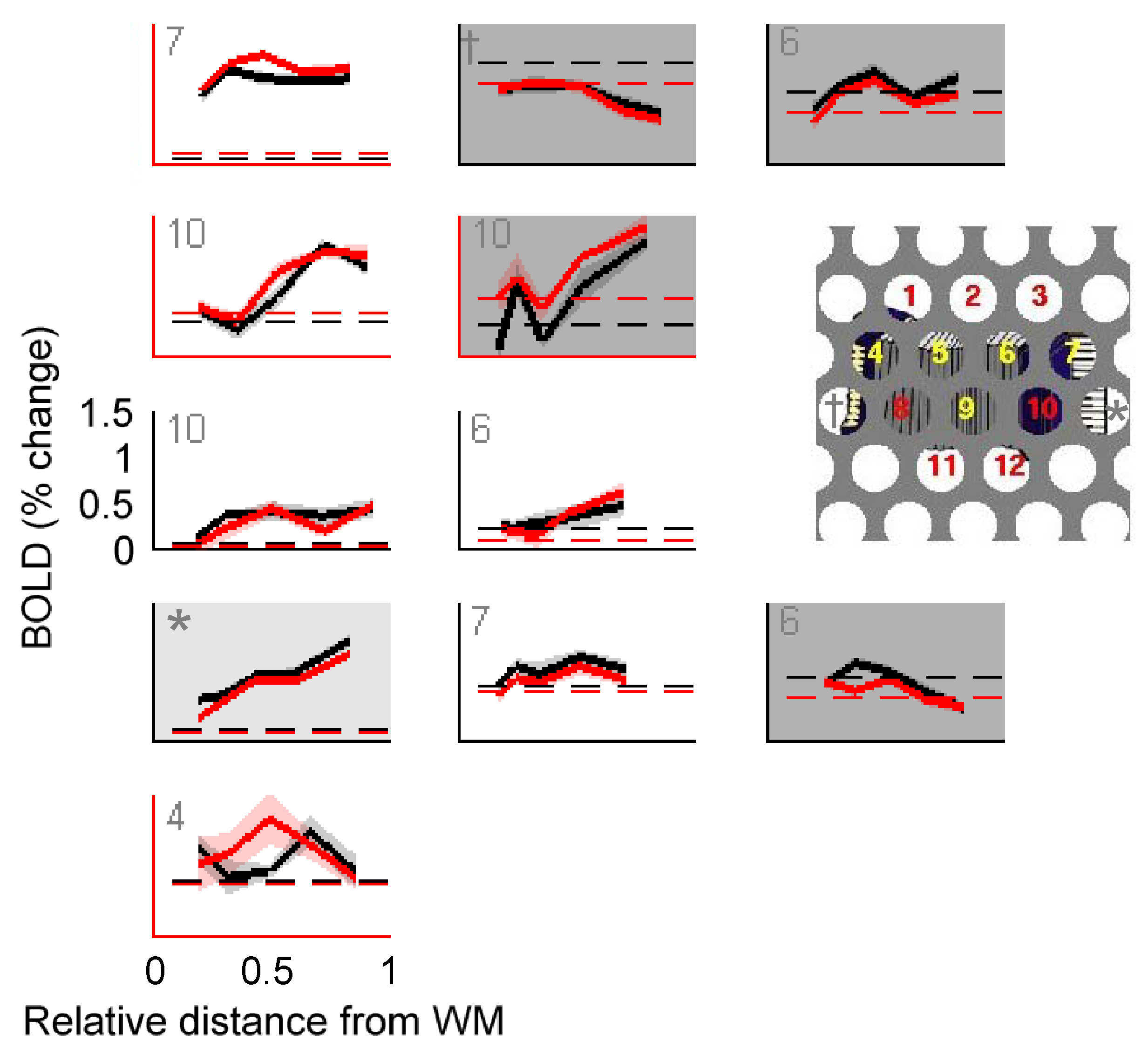

Supplement: Figure S5 — Laminar profiles for all ROIs defined in all subjects. Each row is a different subject, each column is a different ROI. Dark gray backgrounds indicate ROIs excluded due to small size (producing unreliable estimates of BOLD response due to averaging fewer than 40 voxels per bin). Light gray background and/or asterisk and dagger indicate ROIs in a region of the visual field for which visual stimuli were present less than 25% of the time (therefore not included in further analyses). Numbers in upper left of each panel indicate index of ROI, as shown in inset at right (on which yellow numbers indicate ROIs without orientation bias and red numbers indicate ROIs with orientation bias). Color of plot axes indicates whether average response in ROI was stronger for scrambled (red) or intact (black) objects. (TIF) [file pone.0032536.s005.tif]

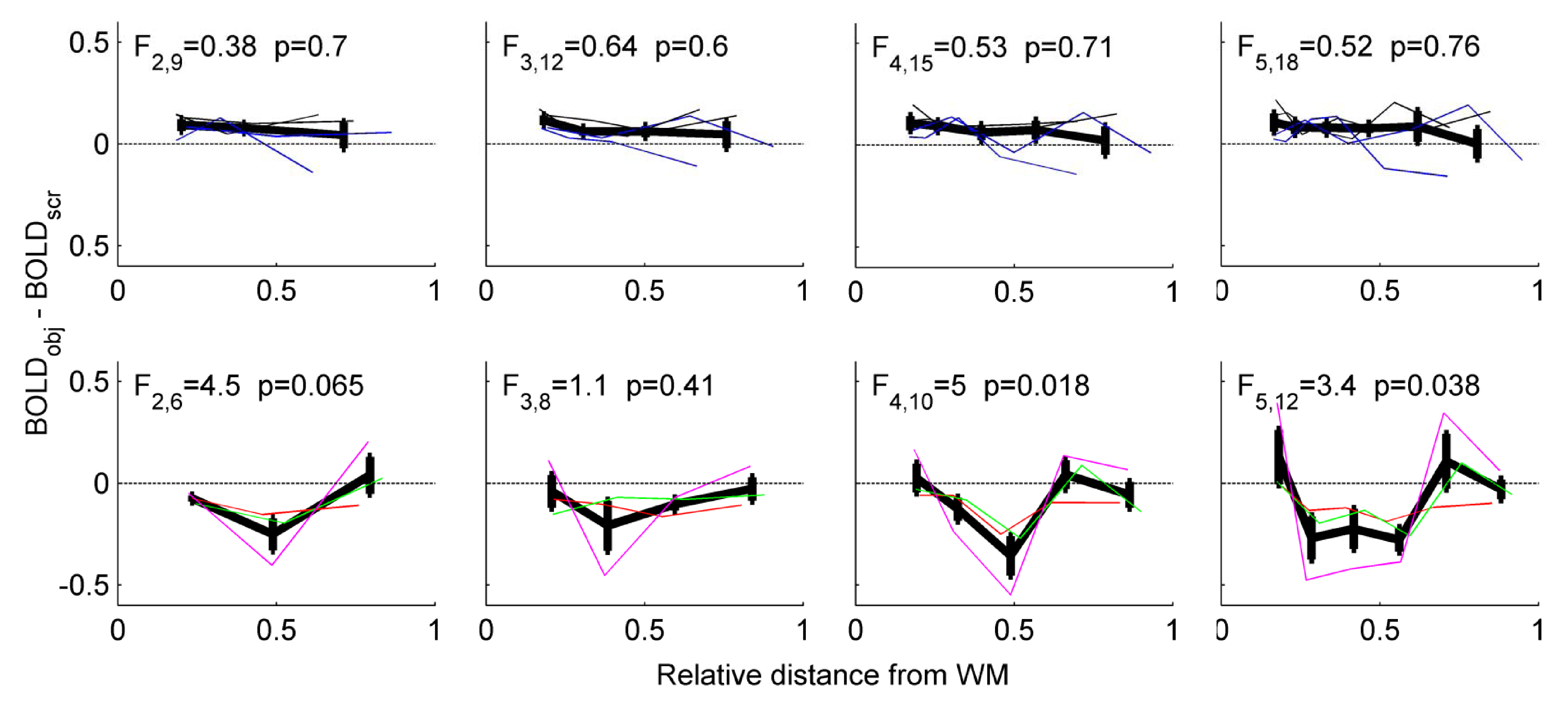

Supplement: Figure S6 — Dependence of laminar profiles on number of bins. Analysis for the main paper used 5 bins (3rd column). Plots in top row are for ROIs showing a larger average response for intact objects; bottom row shows ROIs with stronger average responses to scrambled objects. Text in upper left of each panel indicates F-statistic and p-value from an ANOVA considering the main effect of depth. (TIF) [file pone.0032536.s006.tif]

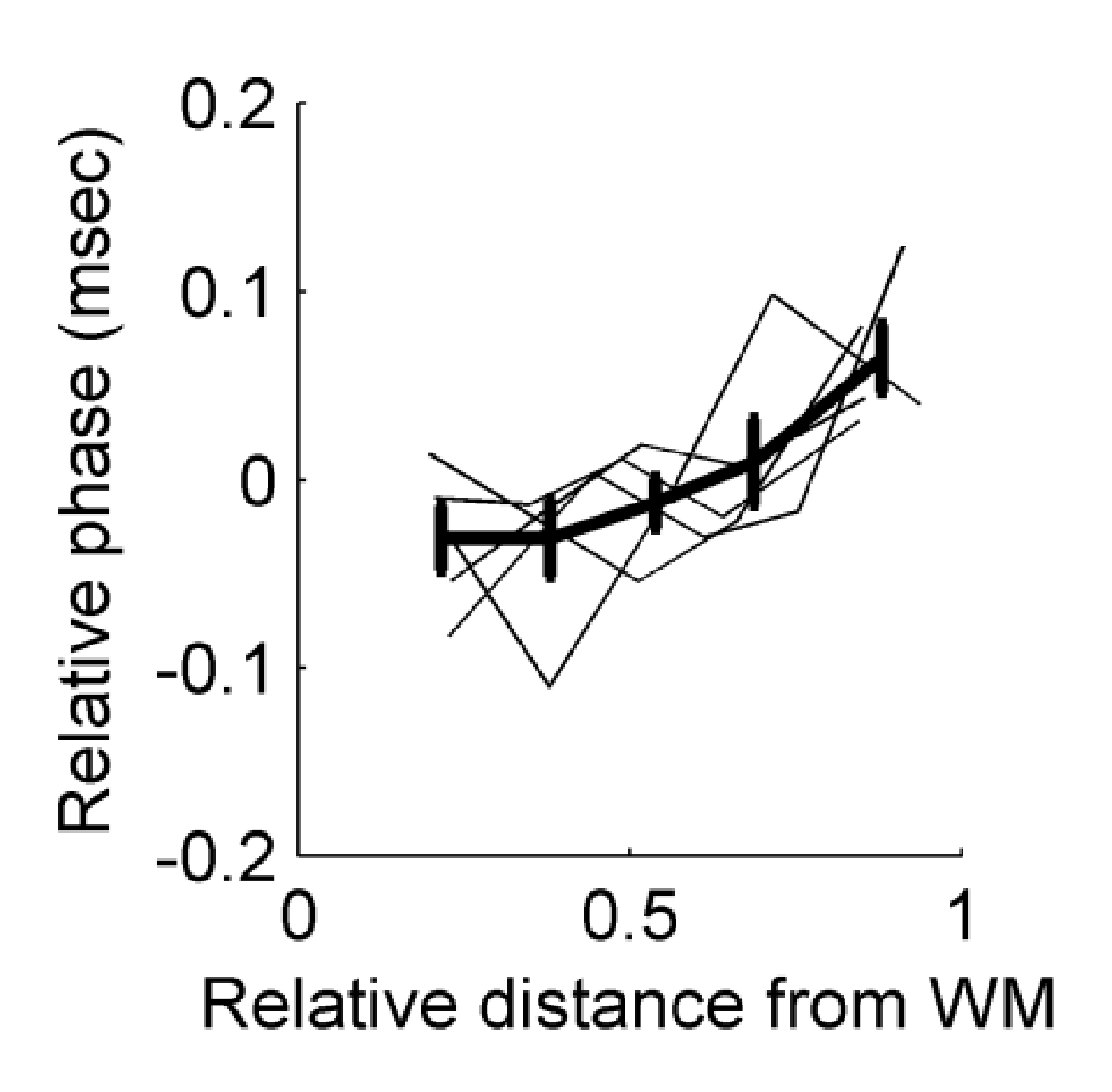

Supplement: Figure S7 — Dependence of onset timing on cortical depth. All scans in a scanning session were averaged together, voxels were divided into five equally-populated depth bins, then for each depth all voxels in a bin were averaged. BOLD response onset was characterized by the phase of the stimulus-related Fourier component at the block-alternation frequency (10 cycles/scan). Stimuli alternated with a 32-second cycle, so the sinusoid phase (a value between 0 and 2π) was scaled to cover the range 0–32 sec. The mean latency was subtracted from the latency profile for each subject (thin black lines) before averaging latencies across subjects. An ANOVA shows a main effect of depth on latency (F4,20 = 4.61, p = 0.0084), consistent with previous reports of shorter onset latencies at the distal extent of penetrating intracortical arterioles. (TIF) [file pone.0032536.s007.tif]
